# UFT/leucovorin and oxaliplatin alternated with UFT/leucovorin and irinotecan in metastatic colorectal cancer

**DOI:** 10.1038/sj.bjc.6602016

**Published:** 2004-07-06

**Authors:** C Alliot

**Affiliations:** 1 Hematology/Oncology Division, General Hospital of Annemasse, BP 525, Annemasse, Cedex 74107, France

**Sir,**

In the January 26, 2004 issue, Petrioli *et al* (2004) reported a small phase II study of 41 patients with metastasic colorectal cancer treated with UFT/leucovorin and oxaliplatin or UFT/leucovorin and irinotecan alternatively. The overall response (58.5%), median progression-free survival (8.8 months) and median overall survival (17.3) months are comparable to those of previous reported combinations including either oxaliplatin or irinotecan (de Gramont *et al*, 2000; Douillard *et al*, 2000; Saltz *et al*, 2000), but with no grade 4 toxicity. Nevertheless, this study is questionable by many aspects. The first point to discuss is the inevitable selection bias illustrated by the favourable general status and the wide predominance (93%) of patients with 1 or 2 metastatic sites. Many baseline characteristics have not been precised such as albumin, lactate dehydrogenase, alkaline phosphatases, or carcinoembryonic antigen levels. Metastasectomy has been performed in about 20% of cases despite very low-dose intensities. This secondary surgery introduces a major confusing factor since prolonged survival and probably cure rates of about 20% can be obtained (Elias *et al*, 1998). Above all, there is a major recruitment bias since 85% of the patients have had been operated for a primary tumour, favouring the early detection of metastases, leading to the selection of patients with low tumour burden and, consequently, to a potential advantage in terms of therapeutic efficacy and tolerance. However, the dose intensities of irinotecan and oxaliplatin are only 36 and 17 mg m^−2^ in this study *vs* 90 and 50 mg m^−2^, respectively, in the FOLFIRI and FOLFOX6 regimens and up to 100 and 65 mg m^−2^ in the recent intensified FOLFIRI-3 (Mabro *et al*, 2003) and FOLFOX7 versions (Maindrault-Goebel *et al*, 2001). UFT also is administered at a dose which is that recommended for combination with a full dose of irinotecan (Alonso *et al*, 2001; Mackay *et al*, 2003). Given the rarity of diarrhoea under oxaliplatin, higher doses of UFT might be combined with this agent (Kim *et al*, 2002).

In fact, the aim of the study may be discussed since efficacy should be privileged in such selected patients potentially candidates to secondary surgery. Possibly, more patients might have benefited from this approach with heavier regimens, taking into account progresses in surgery allowing the treatment of patients with liver and lung metastases (Mineo *et al*, 2003). The question of the selection of resistant clones by low doses also must be addressed. The evaluation of response to either oxaliplatin or irinotecan is extremely difficult with the alternated regimens. Consequently, second-line chemotherapy should have been a dilemma since the investigators had to use mitomycin. This study contributes to demonstrate that, at present, the neoadjuvant approach should be clearly distinguished from palliative chemotherapy. There is also a need for rapid integration of biological predictive factors of response (Etienne *et al*, 2002; Arango *et al*, 2003; Fallik *et al*, 2003; Mariadason *et al*, 2003).

## References

[bib1] Alonso V, Escudero P, Zorrilla M, Isla MD, Herrero A, Mayordomo JI, Martinez-Trufero J, Sáenz A, Tres A, Antón A (2001) Phase I trial of weekly irinotecan combined with UFT as second-line treatment for advanced colorectal cancer. Eur J Cancer 37: 2385–2391 11720832 10.1016/s0959-8049(01)00321-5

[bib2] Arango D, Mariadason JM, Wilson AJ, Yang W, Corner GA, Nicholas C, aranes MJ, Augenlicht LH (2003) c-Myc overexpression sensitises colon cancer cells to camptothecin-induced apoptosis. Br J Cancer 89: 1757–1765 14583781 10.1038/sj.bjc.6601338 PMC2394410

[bib3] De Gramont A, Figer A, Seymour M, Homerin M, Hmissi A, Cassidy J, Boni C, Cortes-Funes H, Cervantes A, Freyer G, Papamichael D, Le Bail N, Louvet C, Hendler D, de Braud F, Wilson C, Morvan F, Bonetti A (2000) Leucovorin and fluorouracil with or without oxaliplatin as first-line treatment in advanced colorectal cancer. J Clin Oncol 18: 2938–2947 10944126 10.1200/JCO.2000.18.16.2938

[bib4] Douillard JY, Cunningham D, Roth AD, Navarro M, James RD, Karasek P, Jandik P, Iveson T, Carmichael J, Alakl M, Gruia G, Awad L, Rougier P (2000) Irinotecan combined with fluorouracil compared with fluorouracil alone as first-line treatment for metastatic colorectal cancer: a multicentre randomised trial. Lancet 355: 1041–1047 10744089 10.1016/s0140-6736(00)02034-1

[bib5] Elias D, Cavalcanti A, Sabourin JC, Pignon JP, Ducreux M, Lasser P (1998) Results of 136 curative hepatectomies with a safety margin of less than 10 mm for colorectal metastases. J Surg Oncol 69: 88–93 9808511 10.1002/(sici)1096-9098(199810)69:2<88::aid-jso8>3.0.co;2-x

[bib6] Etienne MC, Chazal M, Laurent-Puig P, Magné N, Rosty C, Formento JL, Francoual M, Formento P, Renée N, Chamorey E, Bougeon A, Seitz JF, Delpero JR, Letoublon C, Pezet D, Milano G (2002) Prognostic value of tumoral thymidylate synthase and p53 in metastatic colorectal cancer patients receiving fluorouracil-based chemotherapy: phenotypic and genotypic analyses. J Clin Oncol 20: 2832–2843 12065560 10.1200/JCO.2002.09.091

[bib7] Fallik D, Borrini F, Boige V, Viguier J, Jacob S, Miquel C, Sabourin JC, Ducreux M, Praz F (2003) Microsatellite instability is a predictive factor of tumor response to irinotecan in patients with advanced colorectal cancer. Cancer Res 63: 5738–5744 14522894

[bib8] Kim K, Nam E, Lee NS, Lee HR, Lee JY, Lee HR, Park SH, Oh SY, Kim JH, Song SY, Park JO, Kim WS, Jung CW, Im YH, Lee MH, Lee WY, Chun H, Park CH, Park K, Kang WK (2002) Oxaliplatin and UFT combination chemotherapy in patients with metastatic colorectal cancer. Am J Clin Oncol 25: 354–357 12151964 10.1097/00000421-200208000-00007

[bib9] Mabro M, Artru P, Flesch M, Maindrault-Goebel F, André T, Lledo G, Landi B, Aparicio T, Colin P, de Gramont A (2003) Irinotecan, 5-fluorouracil infusion and leucovorin (FOLFIRI-3) in pretreated patients with metastatic colorectal cancer: results of a multicenter phase II study. Proc Am Soc Clin Oncol 22: 280 (abstract 1125)

[bib10] Mackay HJ, Hill M, Twelves C, Glasspool R, Price T, Campbell S, Massey A, Macham MA, Uzzel M, Bailey SM, Martin C, Cunningham D (2003) A phase I/II study of oral uracil/tegafur (UFT), leucovorin and irinotecan in patients with advanced colorectal cancer. Ann Oncol 14: 1264–1269 12881390 10.1093/annonc/mdg340

[bib11] Maindrault-Goebel F, de Gramont A, Louvet C, André T, Carola E, Mabro M, Artru P, Gilles V, Lotz JP, Izrael V, Krulik M (2001) High-dose intensity oxaliplatin added to the simplified bimonthly leucovorin and 5-fluorouracil regimen as second-line therapy for metastatic colorectal cancer (FOLFOX 7). Eur J Cancer 37: 1000–1005 11334725 10.1016/s0959-8049(01)00068-5

[bib12] Mariadason JM, Arango D, Shi Q, Wilson AJ, Corner GA, Nicholas C, Aranes MJ, Lesser M, Schwartz EL, Augenlicht LH (2003) Gene expression profiling-based prediction of response of colon carcinoma cells to 5-fluorouracil and camptothecin. Cancer Res 63: 8791–8812 14695196

[bib13] Mineo TC, Ambrogi V, Tononi G, Bollero P, Roselli M, Mineo D, Nofroni I (2003) Longterm results after resection of simultaneous and sequential lung and liver metastases from colorectal carcinoma. J Am Coll Surg 197: 386–391 12946793 10.1016/S1072-7515(03)00387-9

[bib14] Petrioli R, Sabatino M, Fiaschi AI, Marsili S, Pozzessere D, Messinese S, Correale P, Civitelli S, Tanzini G, Tani F, De Martino A, Marzocca G, Lorenzi M, Giorgi G, Francini G (2004) UFT/leucovorin and oxaliplatin alternated with UFT/leucovorin and irinotecan in metastatic colorectal cancer. Br J Cancer 90: 306–309 14735168 10.1038/sj.bjc.6601521 PMC2409570

[bib15] Saltz L, Cox JV, Blanke C, Rosen LS, Fehrenbacher L, Moore MJ, Maroun JA, Ackland SP, Locker PK, Pirotta N, Elfring GL, Miller LL (2000) Irinotecan plus fluorouracil and leucovorin for metastatic colorectal cancer. N Engl J Med 343: 905–914 11006366 10.1056/NEJM200009283431302

